# Influence of Alcohol and Gender on Immune Response

**Published:** 2002

**Authors:** Elizabeth J. Kovacs, Kelly A.N. Messingham

**Affiliations:** Elizabeth J. Kovacs, Ph.D., is director of the Alcohol Research Program and a professor in the Departments of Cell Biology, Neurobiology and Anatomy, and Surgery, Loyola University Stritch School of Medicine, Maywood, Illinois. Kelly A.N. Messingham, Ph.D., is a postdoctoral research fellow in the Department of Microbiology, University of Iowa, Iowa City, Iowa

**Keywords:** immune response, gender differences, chronic AODE (alcohol and other drug effects), alcoholic beverage, hormones, estrogens, testosterone, cytokines, alcoholic liver disorder, literature review

## Abstract

Decades of research have shown that women’s and men’s immune systems function differently. During the reproductive years, women have a stronger immune response than men. This gender difference is believed to be controlled by differences in the blood levels of gonadal steroid hormones—including the female hormone, estrogen, which stimulates immune responses, and the male hormone, testosterone, which is immunosuppressive. In both males and females, alcohol exposure suppresses immune responses; however, it is unclear whether there are significant gender differences in this suppression. Chronic exposure to alcohol alters the production of this same set of hormones (i.e., estrogen and testosterone), and hence alcohol’s effects on immunity could involve an indirect mechanism in which alcohol alters hormone levels and, in turn, the hormones regulate immune responses. This article discusses evidence that these hormonal changes play a role in the regulation of the immune response following alcohol exposure in males and females. In addition, the article considers the possible reasons why it takes less time and lower doses of alcohol exposure to cause liver damage in females than in males.

Clinical and experimental research has demonstrated naturally occurring gender differences in immune response, but the reasons for these differences have yet to be determined. This article examines alcohol’s effects on the immune systems of both genders and the differential effects of alcohol on males’ and females’ immune responses. It then discusses whether alcohol-induced changes in stress hormones and in gonadal steroid hormones such as estrogen and testosterone are sufficient to trigger the observed defects in immune response and to explain gender differences in alcohol-induced immune suppression. Finally, the article considers the reasons why women are at higher risk than men of developing liver disease at any given level of alcohol intake.

## Alcohol and Immune Responses

An overwhelming amount of evidence reveals that both acute and chronic alcohol exposure suppresses all branches of the immune system, including early responses to infection and the tumor surveillance system (for reviews, see Cook 1998; Diaz et al. 2002; Nelson and Kolls 2002; Messingham et al. 2002). For example, there is a decrease in the ability to recruit and activate germ-killing white blood cells (Deaciuc 1997; Szabo et al. 1999) and an increase in the incidence of breast cancer in people who consume alcohol (Warner-Smith et al. 1998; Zhang et al. 1999).

Some experts suspect that alcohol exerts an “all-or-none” effect on immune response—that is, the presence or absence of alcohol, rather than its amount, dictates the immune response (McGill et al. 1995; Messingham et al. 2002). Other researchers believe that low doses of alcohol—the amount equivalent to a glass of wine—can confer health benefits, including protection against damage to the cardiovascular (Holman et al. 1996) and immune systems (Mendenhall et al. 1997). Such benefits, if they are present, may be attributable to antioxidants in alcoholic beverages such as red wine. In any case, health experts agree that the beneficial effects of antioxidants in some alcoholic beverages are lost if the level of alcohol consumption is elevated (Hanna et al. 1992).

There are several mechanisms by which alcohol impedes immune function. First, alcohol impairs the ability of white blood cells known as neutrophils to migrate to sites of injury and infection, a process called chemotaxis (Bautista 2001). (See the sidebar for a general description of how the immune system works, pp. 261–262) In addition, removing germ-fighting white blood cells (macrophages) and proteins that act as messengers between immune cells (cytokines) from an animal that has not been given alcohol and culturing them in the presence of alcohol, or isolating these cells from humans or animals after administering alcohol, has been shown to alter production of these macrophages and cytokines (Deaciuc 1997; Szabo 1998; Szabo 1999).

**How the Immune System Works**

The immune system is designed to provide protection from invading organisms, including bacteria and viruses, tumor cells, dirt, pollen, and other foreign material. Normally, barriers—including the skin and the lining of the lungs and gastrointestinal and reproductive tracts— protect the underlying delicate tissues from the outside environment. However, when there is a breakdown in that protective lining, germs and other irritants can enter the body. The immune system’s function is to conquer these foreign molecules by engulfing them or by destroying them with enzymes or other detoxifying means. In addition to fighting off these foreign invaders, the immune system has evolved to destroy abnormal cells (such as tumor cells) but occasionally reacts against the body’s own normal tissues (autoimmunity).

***Innate and Acquired Immunity***

There are two principal types of immune response, innate and adaptive (or acquired) immunity, which are distinguished from one another by both their speed and specificity. The innate immune system, so called because it is present from birth, involves nonspecific responses that are the first line of defense against common infectious agents, including bacteria and viruses. This system is generally able to recognize foreign organisms but is unable to distinguish between particular invaders. Thus, an innate response does not require stimulation by sophisticated cell-to-cell interactions to remove bacteria or other foreign material and degrade it.

In contrast to the innate immune system, the more specific adaptive (acquired) immune system must be triggered by a specific virus, bacterium, or other foreign material, which stimulates lymphocytes (see below) to produce antibodies that can combat the foreign substance. At the next exposure, the preformed antibodies will allow the person to respond with an even stronger, more specific response. This is called immunological memory.

***Cells of the Immune System***

The immune system consists of white blood cells (leukocytes), which are produced in the bone marrow and mature there or in the thymus and other lymphoid organs. Leukocytes circulate in the blood along with oxygen-carrying red blood cells. Under normal conditions, leukocytes leave the circulation and migrate into organs, including the skin, lungs, intestine, and reproductive tract, as these are places where germs can appear. There, they can wait for infectious agents, or they can migrate back through the circulation to other organs. There are three major types of leukocytes.

*Neutrophils* are the most plentiful of the white blood cells in humans. They are the immune system’s first line of defense, as they contain an arsenal of preformed chemicals known as enzymes, which are capable of destroying bacteria. In addition, they are phagocytic, meaning that they can engulf viruses, bacteria, or other foreign material, protecting the host from further damage. Neutrophils are very short-lived and are often destroyed during the process of fighting infection.

*Monocytes* are leukocytes that, after migrating to tissues, mature into macrophages. Like neutrophils, macrophages are phagocytic and can remove foreign material and parts of dead cells from the tissues. They too contain enzymes that can destroy infectious material but live longer than neutrophils and do not tend to self-destruct as easily. The tissue macrophage in the liver is called the Kupffer cell.

*Lymphocytes*, the most selective cells of the immune system, are specialized white blood cells that can combat specific infectious agents. There are two types of lymphocytes: B cells and T cells. B cells, which are responsible for humoral immunity (so-called because it takes place in the body fluids, classically known as the humors), release specialized, soluble proteins known as antibodies into the blood and other body fluids. The antibodies recognize and bind to the surface of foreign substances (i.e., pathogens), immobilizing them and further labeling them as foreign so that they can be more readily taken up by phagocytic cells.

T cells, in contrast, act directly on other cells rather than manufacturing antibodies to combat infectious agents. Because of this direct interaction with other cells, T cells are responsible for cellular immunity. They can be further divided into helper T cells, which recognize foreign invaders and stimulate immune responses from other cells; and cytotoxic T cells, which destroy infected cells. Whereas some of these cells survive only briefly, others are extremely long-lived, including “memory cells,” which are capable of remembering certain features on the foreign molecules so that, if the organism encounters that foreign molecule in the future, it can quickly stimulate its response team.

***Communication Between Immune Cells***

One form of communication between immune cells is direct cell-to-cell contact, which can occur either as a loose, transient association or as a tighter, more long-lasting encounter. Either way, cells must make physical contact with one another.

In the second form of contact, cells release small proteins called cytokines, which bind to specific receptors on the surface of target cells. This enables cytokines to interact only with the appropriate target cell with no effect on surrounding cells. Although many of the effects of cytokines are local, they have been called the hormones of the immune system, because like hormones, they are transported by the circulating blood.

Cytokines can affect the same cell that produced them, a neighboring cell, or a cell far away. They stimulate or dampen cell proliferation (replication), production of other cytokines, killing of damaged cells or tumor cells (cytotoxicity), and cell migration (chemotaxis). The latter response is controlled by a subset of cytokines called chemokines. Just as there are cells that can stimulate or inhibit immune response, cytokines produced by those cells can regulate a variety of cell functions either positively or negatively.

—Elizabeth J. Kovacs and Kelly A.N. Messingham

Rodent studies also show that animals are more vulnerable to infection after chronic or acute exposure to alcohol (Deaciuc 1997; Cook 1998; Messingham et al. 2002). This increase in susceptibility is equally dramatic in human patients who sustain traumatic injury (Smith and Kraus 1988; Brezel et al. 1988). Those who have consumed alcohol prior to their injury are six times more likely to die than are alcohol-free patients with comparable injuries (McGill et al. 1995). The mechanisms responsible for this increased mortality are unknown, but it is thought that alcohol compromises the immune system’s ability to quickly fight infection by unidentified invaders—a function of the innate immune system (Faunce et al. 1997; Cook 1998; Messingham et al. 2001).

## Gender Differences in Immune Response Following Alcohol Exposure

To date, only a handful of studies have directly examined gender differences in the effects of alcohol on inflammatory and immune responses (Grossman et al. 1993; Spitzer and Zhang 1996a, b; Lee and Rivier 1996; Li et al. 1998; Spitzer 1999; Spitzer and Spitzer 2000). These studies were conducted in rodents and employed different methods, including varying the quantity and duration of alcohol exposure. These reports show that in the absence of alcohol exposure, inflammatory and immune responses are stronger in females than in males (Grossman et al. 1993; Spitzer and Zhang 1996a, b; Spitzer 1999). However, the increased immunity in females is nullified by alcohol exposure. For example, in one study, proliferation of white blood cells was suppressed in alcohol-exposed female rats (Grossman et al. 1993); however, investigation also showed that alcohol induced an increase in antibody production. In two other studies, female rats were less able to fight infection when intoxicated (Spitzer and Zhang 1996*b*; Li et al. 1998). The mechanisms driving these effects remain uncertain. One possibility is that gender differences in inflammatory and immune responses following alcohol exposure stem from alcohol-induced changes in the production of gonadal steroid hormones, such as estrogen and testosterone.

In general, estrogen stimulates immune responses and testosterone is immunosuppressive (Grossman 1989; Morell 1995; Cannon and St. Pierre 1997; Verthelyi 2001; Burger and Dayer 2002). During their reproductive years, females have more vigorous cellular and humoral immune responses than do males (see the sidebar for a description of these two types of immune response). This heightened immunity in females is evidenced by a more developed thymus,^1^ higher antibody concentrations, and a greater ability to reject tumors and transplanted tissues. Ironically, the enhanced immune function in women of reproductive age is associated with a higher prevalence of autoimmune disorders than is found in postmenopausal women or in men.^2^

The effects of alcohol on production of the gonadal steroid hormones are well documented (Van Thiel et al. 1987; Gavaler and Van Thiel 1992; Gavaler et al. 1993; Gill 2000; Emanuele and Emanuele 2001). In women, chronic alcohol exposure causes an initial increase in estrogen levels, followed by a marked decrease (Gavaler and Van Thiel 1992; Gavaler et al. 1993). In men, chronic alcohol consumption causes a decrease in testosterone (Emanuele and Emanuele 2001). The alcohol-induced decrease in testosterone levels is significant enough to cause shrinkage (atrophy) of the testes, impotence, and loss of secondary sex characteristics (Van Thiel et al. 1987).

### Estrogen and Cytokines

From the limited information available, it is thought that fluctuations in estrogen may alter immune cell function, in part, by increasing or decreasing the production of cytokines (Olsen and Kovacs 1996; Cannon and St. Pierre 1997; Verthelyi 2001; Burger and Dayer 2002). There are several pieces of evidence for this idea. First, researchers found that removing the ovaries of adult rodents (eliminating the primary source of estrogen) lowered the level of cytokine production by certain types of white blood cells (Frazier-Jessen and Kovacs 1995; Chao et al. 1995; D’Agostino et al. 1999; Deshpande et al. 1997). This lower level of cytokine production was comparable to that of males and could be restored by administering estrogen (Gregory et al. 2000*a*).

In other studies, drugs known as estrogen receptor antagonists inhibited the effect of estrogen on immune cells in animals (Gregory et al. 2000*b*; Wu et al. 1999). While receptor antagonists are bound to the same receptors that normally interact with estrogen, they block the binding of the hormone. Thus, it is possible to alter immune responses by blocking estrogen at one of its sites of action in white blood cells.

Further evidence that estrogen affects immune cell function, in part, by altering production of cytokines comes from cell-culture studies in which estrogen was added to a culture of white blood cells (Burger and Dayer 2002). The effects of estrogen on cytokine production by immune target cells may involve direct interaction (binding) of the hormone and hormone receptors within those cells (Hall et al. 2001). The idea of direct effects of estrogen on target cells is supported by the existence of estrogen receptors not only in reproductive tissues, including the uterus, ovaries, and testes, where one would expect the hormone’s actions to occur, but also in white blood cells (Weusten et al. 1986; Gulshan et al. 1990; Benten et al. 2001).

### Alcohol, Stress Responses, and Immunity

**Figure 1:**
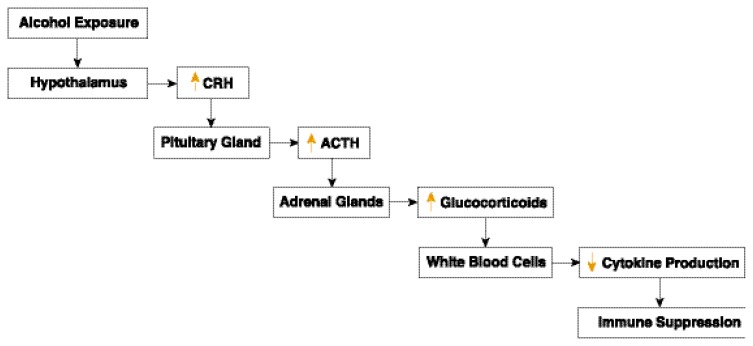
Involvement of the hypothalamic–pituitary–adrenal axis in alcohol-induced immune suppression. Alcohol exposure stimulates the hypothalamus to produce corticotropin-releasing hormone (CRH). This triggers the pituitary gland to secrete adrenal corticotropic hormone (ACTH), which in turn stimulates the adrenal glands to release glucocorticoids. At high levels, glucocorticoids signal white blood cells to alter cytokine production, suppressing inflammatory and immune responses. (Yellow arrows indicate increased or decreased production or activity.)

**Figure 2:**
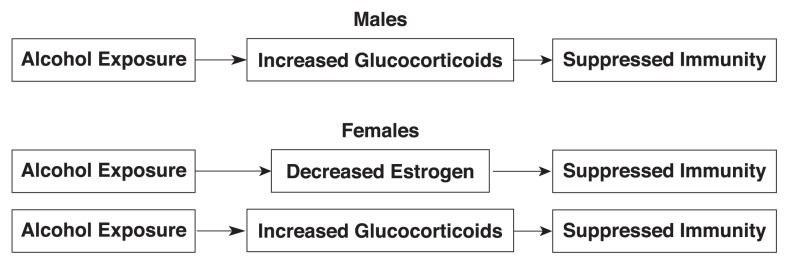
Hormone-dependent pathways by which alcohol could suppress immune responses. As the figure illustrates, there is only one potential pathway in males, but there are two in females, which could result in an additive effect.

Like other stressors, alcohol stimulates a neuroendocrine network known as the hypothalamic–pituitary–adrenal (HPA) axis, resulting in a dampening of the immune response (Eskandari and Sternberg 2002) (see figure 1). This process begins with activation of the hypothalamus (near the base of the brain), which produces a molecule called corticotropin-releasing hormone (CRH). This triggers the pituitary gland (below the hypothalamus) to secrete adrenal corticotropic hormone (ACTH). Finally, ACTH stimulates the adrenal glands (above the kidneys) to release glucocorticoids (cortisol in humans and corticosterone in rodents). These steroid hormones, which direct the activity of many cell types, are transmitted throughout the body in the blood. At high levels, they suppress inflammatory and immune responses (Guyre and Goulding 1993; Da Silva 2002). Several studies have documented that under resting (baseline) conditions and in response to stress, females have higher levels of glucocorticoids than do men (Kant et al. 1983; Chasari et al. 1995). Furthermore, estrogen stimulates glucocorticoid production in females (Burgess and Handa 1992; Carey et al. 1995), whereas testosterone suppresses its production in both male and female subjects (Carlstrom and Stege 1990; De Weerdt and Gooren 1992; Handa et al. 1994). Alcohol exposure stimulates glucocorticoid production in both males and females (Ogilvie et al. 1998; Eskandari and Sternberg 2002). Thus, there are two possible pathways by which alcohol-induced changes in steroid hormones could suppress immune responses in females, whereas there is only one such potential pathway in males (as shown in figure 2). Further study will be required to determine if and how the two pathways interact to mediate alcohol-induced effects on immune function in females.

### Gender, Alcohol, and Liver Damage

Epidemiologic evidence clearly indicates that the adverse consequences of alcohol consumption, including severe liver disease, such as alcoholic cirrhosis, develop more quickly and require lower levels of alcohol exposure for females than for males (Ashley et al. 1977; Loft et al. 1987; Schenker 1997; for reviews, see Thurman 2000; Diehl 2002). At any given level of alcohol intake, women are at higher risk than men of developing liver disease (Ashley et al. 1977; Loft et al. 1987; Dawson 1994; Schenker 1997). It has been shown that a daily alcohol ingestion of as low as two drinks per day increases the risk of developing cirrhosis in women, although at least four drinks per day are required to increase this risk in men (Ashley et al. 1977; Loft et al. 1987; Thomasson 1995; Frezza et al. 1990; Taylor et al. 1996). These observations were made taking into account differences in body weight, fat distribution, body water, and other potentially confounding variables.

The mechanisms responsible for the gender difference in alcohol-related liver injury are currently under intense investigation and have been better described in animal studies (Kono et al. 2000; Nanji et al. 2001). Performing studies in animals allows the investigator to include experiments involving hormone manipulations that would not be feasible in human experimentation. These experiments could include removing ovaries (the primary site of estrogen production) or giving a hormone receptor antagonist (i.e., a molecule that blocks the hormone from binding to its receptor).

It is possible that gender differences in alcohol-related liver disease could be explained by gender differences in:

- The breakdown and elimination of alcohol and its byproducts, including the resulting differences in acetaldehyde levels within the liver (Thomasson 1995; Li et al. 2000).
- The level of activation of inflammatory and immune cells within the liver in response to alcohol ingestion, including Kupffer cells (Adachi et al. 1994; Kono et al. 2000; Nanji et al. 2001; McClain et al. 2002).^3^ Upon stimulation, these cells produce free oxygen radicals and cytokines, which damage and destroy liver cells (Adachi et al. 1994).
- The amount of alcohol that is metabolized in the stomach (first-pass metabolism). Some research has indicated that women break down less alcohol in the stomach than men do, leading to higher blood alcohol levels—and hence greater risk to the liver—for a given dose of alcohol (Pozzato et al. 1995; Baraona et al. 2001).

## Summary

Taken together, these studies show clearly that there are dramatic suppressive effects of both acute and chronic alcohol exposure on inflammation and immunity, regardless of gender. This results in decreased ability of the immune system to fight infections and tumors. The decrease in immunity after consumption of larger quantities of alcohol is in marked contrast to the effects of very low levels of some alcoholic beverages (such as a single glass of red wine), which contain immunoprotective antioxidants. By depressing estrogen levels, chronic or acute alcohol exposure may cause females to lose the important boost to the immune system that estrogen normally provides. This could act additively or synergistically with an elevation in immunosuppressive glucocorticoids (through activation of the HPA axis) to attenuate immune response, thus leading to a weakened ability to fight infections and tumors. Finally, although chronic alcohol exposure causes liver damage in both males and females, it takes less alcohol and shorter periods of consumption to raise the risk of liver damage for females than for males. Like the observed gender differences in alcohol-induced immune suppression, this effect may involve the combined effect of stimulating glucocorticoid production and inhibiting estrogen production (see figure 2).

Further studies will be required to determine whether the alcohol-induced changes in gonadal steroid hormone production are sufficient to explain the observed gender differences in immune function. These will require using a similar model system in which both males and females are given alcohol at doses designed to raise blood alcohol levels to the same extent, after which immune responses can be examined. Because of the complexity of studying these parameters in humans, it may be necessary to conduct these studies in animal models of alcohol exposure. By using animal models, it will also be possible to manipulate the levels of estrogen, testosterone, and glucocorticoids by removing organs (ovaries, testes, and adrenal glands, respectively) and administering hormones and hormone antagonists to determine the role of those hormones in regulating inflammatory and immune responses after alcohol exposure.

## Footnotes

1 The thymus is a gland located in the upper chest that is involved in the maturation of immune cells.

2 Although estrogen is present in males, its concentration is too low to affect immune response.

3 Kupffer cells are star-shaped immune cells that reside in the microscopic blood vessels of the liver. They are phagocytic, meaning that they are capable of ingesting other cells and foreign particles. Alcohol exposure can cause leakiness of the digestive tract, which can result in the release of endotoxins (bacterial cell wall products) from the gut into the blood. These endotoxins travel through the blood to the liver, where they can activate Kupffer cells.
